# Saturation effect of brachial-ankle pulse wave velocity on first stroke in adults with hypertension: a prospective cohort study

**DOI:** 10.3389/fcvm.2025.1535366

**Published:** 2025-09-03

**Authors:** Wei Zhou, Lingjuan Zhu, Tao Wang, Chao Yu, Huihui Bao, Xiaoshu Cheng

**Affiliations:** ^1^ Center for Prevention and Treatment of Cardiovascular Diseases, The Second Affiliated Hospital of Nanchang University, Nanchang, Jiangxi, China; ^2^ Jiangxi Provincial Cardiovascular Disease Clinical Medical Research Center, Nanchang, Jiangxi, China; ^3^ Department of Cardiovascular Medicine, The Second Affiliated Hospital of Nanchang University, Nanchang, Jiangxi, China

**Keywords:** brachial-ankle pulse wave velocity, stroke, hypertension, saturation effect, elderly

## Abstract

**Background:**

Previous studies have reported a linear association between brachial-ankle pulse wave velocity (baPWV) and stroke in hypertensive individuals, primarily in foreign countries, with few studies conducted in China. This study aimed to investigate the saturation effect of baPWV on the first stroke in adults with hypertension and propose a possible inflection point of baPWV at which the saturation effect occurs.

**Methods:**

A total of 7,198 adults with hypertension and baseline baPWV were enrolled from the China Hypertension Registry Study. The outcome of this study was the first stroke. Cox proportional hazards regression, smoothing curve fitting (restricted cubic spline), Kaplan–Meier survival curve analysis, and subgroup analysis were used to investigate the association between baPWV and first stroke.

**Results:**

A total of 281 patients experienced their first stroke during an average of four years of follow-up. There was a saturation effect of baPWV with an inflection value of 17.5 m/s on the first stroke. For baPWV < 17.5 m/s, each 1 m/s increment was associated with a 31% higher risk of first stroke (hazard ratio [HR]: 1.31, 95% confidence interval [CI]: 1.17, 1.47). For baPWV ≥ 17.5 m/s, there was no significant association between baPWV and first stroke (HR: 0.99, 95% CI: 0.96, 1.02) (for log-likelihood ratio test *P* < 0.001). Kaplan–Meier curves revealed a continual increase in the cumulative hazard for the first stroke from quartile 1–3 levels of baPWV (log-rank *P* < 0.001), whereas a non-significant difference in cumulative hazard between quartiles 3 and 4 was observed (log-rank *P* = 0.873).

**Conclusion:**

BaPWV exhibited a saturation effect on the first stroke in hypertensive adults in China. Increased baPWV was positively associated with a higher risk of first stroke among hypertensive adults with a baPWV < 17.5 m/s.

## 1 Introduction

Stroke remains a leading cause of disability and mortality in China, imposing a substantial socioeconomic burden that has escalated steadily over the past three decades (1, 2). Although primary prevention and early intervention are recognized as optimal strategies for mitigating stroke-related morbidity and economic costs (3), the insufficient explanatory power of traditional risk factors in stroke pathogenesis highlights the need to investigate novel biomarkers of vascular dysfunction (4).

Emerging evidence positions arterial stiffness as a reliable feature of arterial structure and function (5), with proven prognostic value in hypertensive populations (6, 7). The European guidelines for the management of hypertension introduce the evaluation of arterial stiffness by pulse wave velocity (PWV) as a measure of cardiovascular target organ damage associated with hypertension. PWV is a relatively simple, noninvasive, and reproducible measurement for assessing arterial stiffness and is most widely used in clinical practice. Numerous studies have proven the accuracy of PWV as an independent predictor of cardiovascular events and mortality (8). Currently, the most commonly used indicators of PWV are carotid-femoral pulse wave velocity (cfPWV) and brachial ankle pulse wave velocity (baPWV). A study comparing aortic PWV and baPWV revealed that baPWV exhibited excellent validity and reproducibility and was an acceptable marker of vascular damage (9). Additionally, cfPWV is known as the gold standard for measuring arterial stiffness; however, its time-consuming nature and high technical requirements render it unsuitable for large-scale research. Therefore, baPWV is used as an alternative measurement of arterial stiffness (10). BaPWV exhibited stronger correlations with cardiovascular hemodynamics and coronary calcification than cfPWV (11, 12). Although international studies have reported a linear relationship between baPWV elevation and stroke incidence in hypertensive individuals (7, 13–15), critical knowledge gaps persist regarding population-specific risk patterns in China's unique healthcare context and potential threshold effects of baPWV values on disproportionate stroke risk.

To address these uncertainties, this prospective cohort study investigated the association between baPWV and first stroke in the Chinese hypertensive population, with particular emphasis on identifying potential nonlinear relationships.

## 2 Methods

### 2.1 Study participants

This study was a subset of the China Hypertension Registry Study (Registration website: http://www.chictr.org.cn/, Number: ChiCTR1800017274, Date: July 20, 2018), a real-world, prospective, and observational study conducted in Wuyuan, Jiangxi Province, China, from March 2018 to August 2018. The methodology for data acquisition and exclusion criteria has been previously described (16, 17). This study adhered to the Declaration of Helsinki and was approved by the Ethics Committee of the Second Affiliated Hospital of Nanchang University (Ethics No. 2018019) and the Institute of Biomedical Sciences, Anhui Medical University (Ethics No. CH1059). Written informed consent was obtained from all enrolled participants.

**Figure 1:**
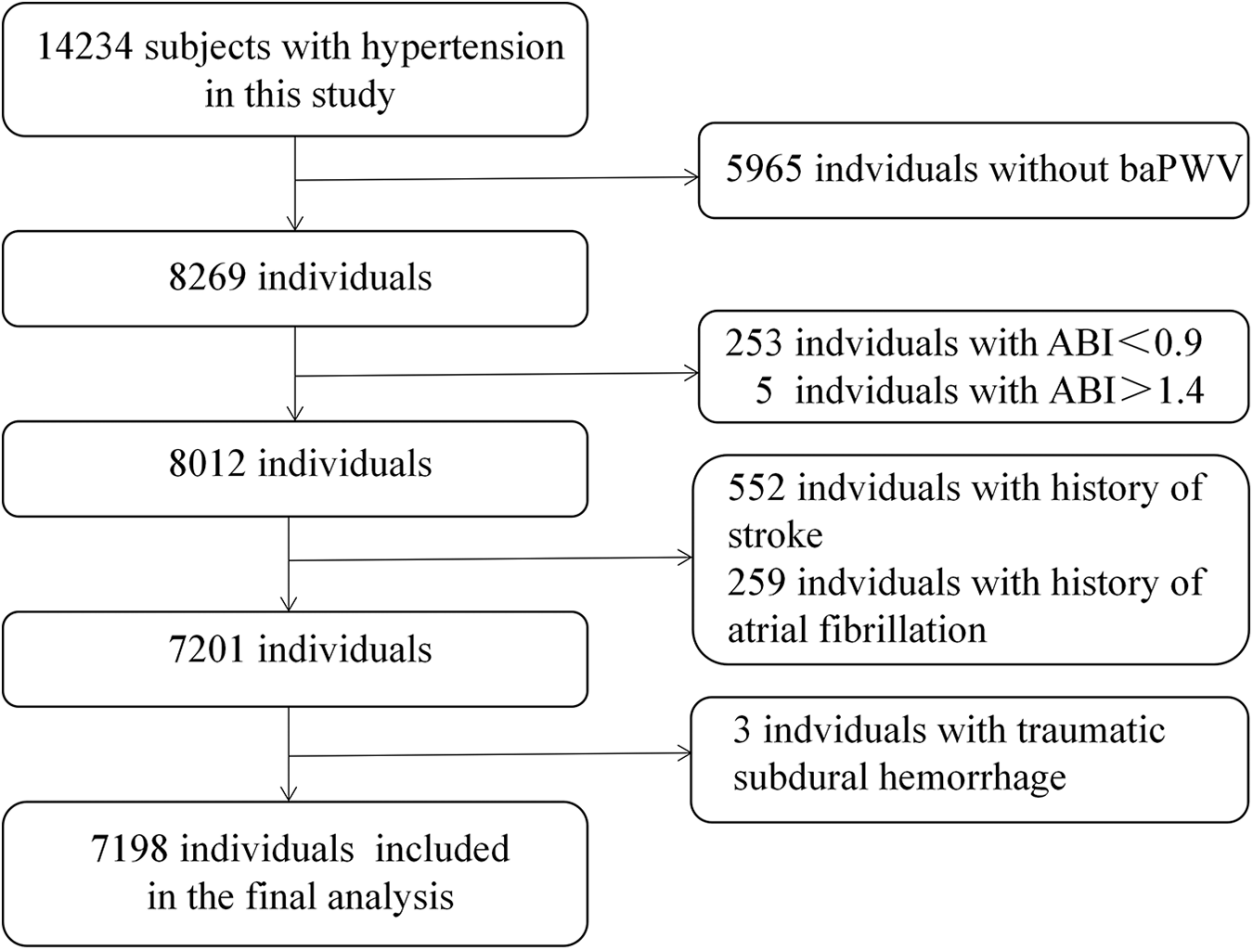
Flowchart of the study participants.

From the initial cohort of 14,234 hypertensive individuals recruited at baseline, we sequentially excluded individuals lacking baPWV values (*n* = 5,965) and those with ankle-brachial index (ABI) <0.90 (*n* = 253), ABI > 1.4 (*n* = 5), stroke (*n* = 552), atrial fibrillation (*n* = 259) and traumatic subdural hemorrhage (*n* = 3). Finally, this prospective study included 7,198 participants for analysis. The selection process for the analytic sample is presented in Figure 1.

### 2.2 Covariates

Based on the existing literature and clinical applications, covariates obtained from physical examinations, standard questionnaires, and laboratory measurements were selected as potential confounders. Physical examinations and standard questionnaires were administered by trained staff following a standard operating procedure. A fully adjusted model was developed using the following covariates. Categorical variables included gender, current smoking, current drinking, sleep duration, previous medical diagnoses (hypertension, dyslipidemia, diabetes, coronary heart disease, heart failure, atrial fibrillation, and stroke), and current medication based on drug packaging (antihypertensive, lipoprotein-lowering, glucose-lowering, and antiplatelet drugs). Continuous variables included age, body mass index (BMI), systolic blood pressure (SBP), diastolic blood pressure (DBP), homocysteine (Hcy), total cholesterol (TC), triglyceride (TG), high-density lipoprotein cholesterol (HDL-C), and low-density lipoprotein cholesterol (LDL-C). The BMI was calculated as weight (kg)/height (m^2^). Blood pressure was measured thrice using an electronic sphygmomanometer on the right arm positioned at the heart level after a 5-min rest, with a 30-s interval between measurements. The average of the three measurements was used.

### 2.3 BaPWV measurement

The automatic device of Omron-Colin BP-203RPE (Omron Health Care, Japan) was used to measure simultaneously baPWV and ankle-brachial index (ABI). After resting in the supine position for more than 5 min, four cuffs were wrapped around bilateral brachia and ankle arteries of participants, and connected to a plethysmographic sensor and oscillometric pressure sensor. Pulse volume waveform were recorded using semiconductor pressure sensors to assess the time interval between the initial increase in brachial and tibial waves.

The ABI value of each leg was calculated by the ankle SBP divided by the ipsilateral brachial SBP, and the lower value in two legs was used in the analysis. The baPWV was calculated by (La-Lb)/Tba. La is the path distance between the suprasternal notch and the ankle, Lb is the path distance between the suprasternal notch and the brachium, and Tba is the time interval between the brachial and ankle waveform. The larger values of baPWV on the left and right sides were used for the main analysis (18), and the average values on both sides were used for a sensitivity analysis.

### 2.4 Outcome assessment

Details on the definition and event adjudication were sourced from the literature (19). Stroke event identification followed a rigorous, multistep protocol. First, potential cases were preliminarily identified using retrospective patient interviews, medical record reviews (including hospitalizations and emergency department visits), and linkage with the national health insurance database. Subsequently, all suspected cases were systematically verified by retrieving and analyzing neuroimaging records (for instance, CT/MRI scans). Finally, confirmed cases were reviewed and adjudicated through independent discussion by an Endpoint Adjudication Committee comprising neurologists, neurosurgeons, and public health experts.

The primary outcome was the first occurrence of fatal or nonfatal symptomatic stroke (ischemic or hemorrhagic), excluding subarachnoid hemorrhage and silent strokes. Participants with preexisting stroke at baseline were excluded. Recurrent strokes were documented but not considered primary outcomes. The follow-up period was extended from the baseline survey completion date to August 15, 2022.

Patients with a history of stroke at baseline were excluded. The first attack of symptomatic stroke was considered the first stroke in the patient unless there was evidence against it. Any stroke after the first attack was considered a recurrent stroke, which was not the primary outcome of this study.

### 2.5 Statistical analysis

Continuous variables are presented as mea*n* ± standard deviation (SD), and categorical variables are presented as frequencies and percentages (%). Differences in baseline characteristics according to baPWV categories were compared using a one-way analysis of variance for continuous variables and a chi-square test for categorical variables. Cox proportional hazard regression was used to evaluate the HRs and 95% CIs for the association between baPWV and first stroke, with adjustment for potential covariates in the three models. The proportional hazards assumption was evaluated using Schoenfeld residual tests, which revealed no violations (*P* > 0.05), indicating that the assumption of proportionality was satisfied. Potential covariates adjusted in the models were included due to their clinical importance, statistical significance in the univariable analysis, and the potential confounder effect estimates being individually changed by at least 10%. The cumulative hazards of the first stroke by baPWV categories were estimated using the Kaplan–Meier curve, and differences between groups were compared using the log-rank test. The generalized additive model and a restricted cubic spline were used to characterize the dose-response association of baPWV with the first stroke. Additionally, potential modifications to the association between baPWV and first stroke were evaluated using subgroup analyses.

A two-tailed *P* < 0.05 was considered statistically significant. The R statistical package (version 4.2.3; http://www.r-proje.ct.org) and Empower (R) software (version 4.1; http://www.empow.erstats.com) were used for statistical analyses.

## 3 Results

### 3.1 Baseline characteristics

This study included 7,198 individuals with hypertension [mean age, 63.7 (9.7) years], and 47.1% of them were male. The mean ± SD value of baseline baPWV was 18.2 ± 5.4 m/s. The baseline characteristics of all participants stratified by the baPWV quartile are listed in Table 1. Participants with a higher baPWV were more likely to be female and have a higher age, SBP, DBP, Hcy, TC, HDL-C, and LDL-C levels, and a lower BMI. Additionally, they exhibited a longer sleep duration of <5 and >8 h, use of antihypertensive and glucose-lowering drugs, a history of diabetes and congenital heart defect (CHD), a lower rate of current smoking and current drinking, and a history of dyslipidemia (*P* < 0.05).

**Table 1:** Baseline characteristics of participants stratified by baPWV.

Characteristics	Total	Quartiles of baPWV (m/s)				*P*
		Q1 (5.6–15.0)	Q2 (15.1–17.0)	Q3 (17.1–19.9)	Q4 (20.0–64.8)	
N	7198	1,799	1,799	1,799	1,801	
Age, y	63.73 ± 9.66	58.14 ± 9.13	62.77 ± 8.81	65.26 ± 8.70	68.75 ± 8.77	<0.001
Male, *n* (%)	23.53 ± 3.53	990 (55.03)	868 (48.20)	787 (43.75)	748 (41.53)	<0.001
BMI (kg/m^2^)	146.82 ± 17.10	24.06 ± 3.50	23.75 ± 3.49	23.42 ± 3.54	22.87 ± 3.47	<0.001
SBP (mmHg)	88.93 ± 10.76	137.42 ± 14.02	144.12 ± 14.58	149.74 ± 15.69	155.99 ± 18.05	<0.001
DBP (mmHg)	17.84 ± 10.91	88.05 ± 9.96	88.30 ± 10.42	88.63 ± 10.82	90.74 ± 11.58	<0.001
Hcy (mmol/L)	5.09 ± 1.11	17.12 ± 10.81	17.44 ± 9.84	17.96 ± 11.20	18.84 ± 11.62	<0.001
TC (mmol/L)	1.81 ± 1.28	4.97 ± 1.10	5.01 ± 1.11	5.16 ± 1.11	5.22 ± 1.11	<0.001
TG (mmol/L)	1.52 ± 0.40	1.76 ± 1.26	1.81 ± 1.25	1.84 ± 1.41	1.83 ± 1.20	0.272
HDL-C (mmol/L)	2.94 ± 0.77	1.50 ± 0.39	1.51 ± 0.41	1.54 ± 0.41	1.54 ± 0.40	0.007
LDL-C (mmol/L)	63.73 ± 9.66	2.90 ± 0.76	2.90 ± 0.77	2.97 ± 0.76	2.99 ± 0.78	<0.001
Current smoking, *n* (%)	1,990 (27.65)	557 (30.96)	546 (30.32)	475 (26.40)	412 (22.88)	<0.001
Current drinking, *n* (%)	1,701 (23.63)	485 (26.96)	440 (24.43)	408 (22.68)	368 (20.43)	<0.001
Sleep duration, *n* (%)						<0.001
<5 h	297 (4.12)	56 (3.11)	71 (3.94)	81 (4.50)	89 (4.94)	
5–8 h	3,704 (51.46)	1,041 (57.87)	900 (49.97)	913 (50.75)	850 (47.21)	
>8 h	3,197 (44.42)	702 (39.02)	830 (46.09)	805 (44.75)	860 (47.75)	
Antihypertensive drugs, *n* (%)	4,357 (60.51)	1,136 (63.15)	1,066 (59.19)	1,057 (58.75)	1,098 (60.97)	0.029
Glucose-lowering drugs, *n* (%)	310 (4.31)	54 (3.00)	71 (3.94)	85 (4.72)	100 (5.55)	0.001
Lipoprotein-lowering drugs, *n* (%)	180 (2.50)	40 (2.22)	59 (3.28)	45 (2.50)	36 (2.00)	0.076
Antiplatelet drugs, *n* (%)	161 (2.24)	32 (1.78)	42 (2.33)	42 (2.33)	45 (2.50)	0.485
Diabetes, *n* (%)	1,283 (17.82)	237 (13.17)	297 (16.49)	352 (19.57)	397 (22.04)	<0.001
Coronary heart disease, *n* (%)	394 (5.47)	76 (4.22)	92 (5.11)	107 (5.95)	119 (6.61)	0.011
Heart failure, *n* (%)	74 (1.03)	25 (1.39)	14 (0.78)	15 (0.83)	20 (1.11)	0.239
Dyslipidemia, *n* (%)	1,187 (16.49)	339 (18.84)	309 (17.16)	279 (15.51)	260 (14.44)	0.002
First stroke, *n* (%)	281 (3.90)	33 (1.83)	61 (3.39)	92 (5.11)	95 (5.27)	<0.001

BMI, body mass index; SBP, systolic blood pressure; DBP, diastolic blood pressure; Hcy, homocysteine; TC, total cholesterol; TG, triglycerides; HDL-C, high-density lipoprotein cholesterol; LDL-C, low-density lipoprotein cholesterol; CHD, coronary heart disease.

### 3.2 Association of baPWV with first stroke

During an average follow-up period of four years, 281 cases were identified with the first stroke. The independent effects of baPWV on the first stroke are illustrated in Table 2. After adjusting for potential confounders in model 3, each 1 m/s increment of baPWV was associated with a 2% increase in the risk of the first stroke (HR: 1.02; 95% CI: 1.01, 1.04). When baPWV was measured in quartiles, the HRs (95% CIs) of the first stroke for participants in quartiles 1, 2, and 4 were 0.39 (0.26, 0.59), 0.69 (0.50, 0.96), and 1.00 (0.74, 1.33) respectively, compared with those in quartile 3 (*P* for trend = 0.810) (Table 2).

**Table 2:** Association between baPWV and first stroke.

baPWV, m/s	N	Events, *n* (%)	Model 1		Model 2		Model 3	
			HR (95% CI)	*P*	HR (95% CI)	*P*	HR (95% CI)	*P*
Per 1 m/s increase	7,198	281 (3.9)	1.02 (1.01, 1.04)	<0.001	1.02 (1.01, 1.04)	0.002	1.02 (1.01, 1.04)	0.025
Quartiles								
Q1 (<15.0)	1,799	33 (1.8)	0.35 (0.24, 0.52)	<0.001	0.37 (0.24, 0.55)	<0.001	0.39 (0.26, 0.59)	<0.001
Q2 (15.1–17.0)	1,799	61 (3.4)	0.66 (0.48, 0.91)	0.012	0.66 (0.48, 0.91)	0.012	0.69 (0.50, 0.96)	0.026
Q3 (17.1–19.9)	1,799	92 (5.1)	1.00		1.00		1.00	
Q4 (≥20.0)	1,801	95 (5.3)	1.05 (0.79, 1.40)	0.722	1.04 (0.78, 1.38)	0.810	1.00 (0.74, 1.33)	0.970
*P* for trend			0.260		0.452		0.810	

Model 1 was adjusted for none.

Model 2 was adjusted for gender and age.

Model 3 was adjusted for gender, age, BMI, SBP, DBP, Hcy, TC, TG, HDL-C, LDL-C, current smoking, current drinking, sleep duration, antihypertensive drugs, glucose-lowering drugs, lipid-lowering drugs, antiplatelet drugs, diabetes, CHD, heart failure, and dyslipidemia.

HR, hazard ratio; CI, confidence interval; LLR, log-likelihood ratio; BMI, body mass index; SBP, systolic blood pressure; DBP, diastolic blood pressure; Hcy, homocysteine; TC, total cholesterol; TG, triglycerides; HDL-C, high-density lipoprotein cholesterol; LDL-C, low-density lipoprotein cholesterol; CHD, coronary heart disease.

### 3.3 Saturation effect of baPWV on first stroke

**Figure 2:**
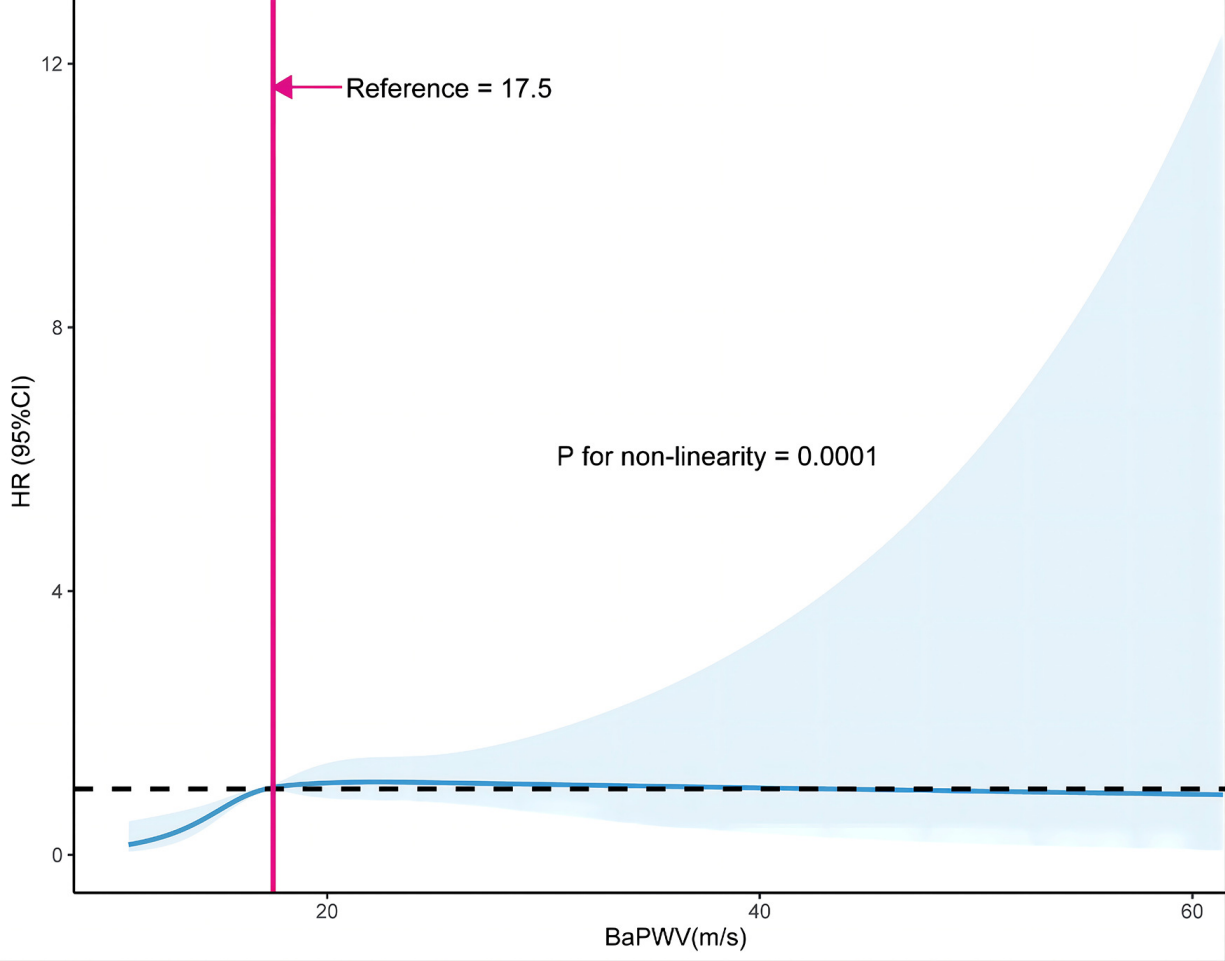
Restricted cubic spline curve of the association between baPWV and first stroke. The solid and dashed lines represent the estimated values and their corresponding 95% CI. Adjustment factors included gender, age, BMI, SBP, DBP, Hcy, TC, TG, HDL-C, LDL-C, current smoking, current drinking, sleep duration, antihypertensive drugs, glucose-lowering drugs, lipid-lowering drugs, antiplatelet drugs, diabetes, CHD, heart failure, and dyslipidemia. BMI, body mass index; SBP, systolic blood pressure; DBP, diastolic blood pressure; Hcy, homocysteine; TC, total cholesterol; TG, triglycerides; HDL-C, high-density lipoprotein cholesterol; LDL-C, low-density lipoprotein cholesterol; CHD, coronary heart disease.

**Figure 3:**
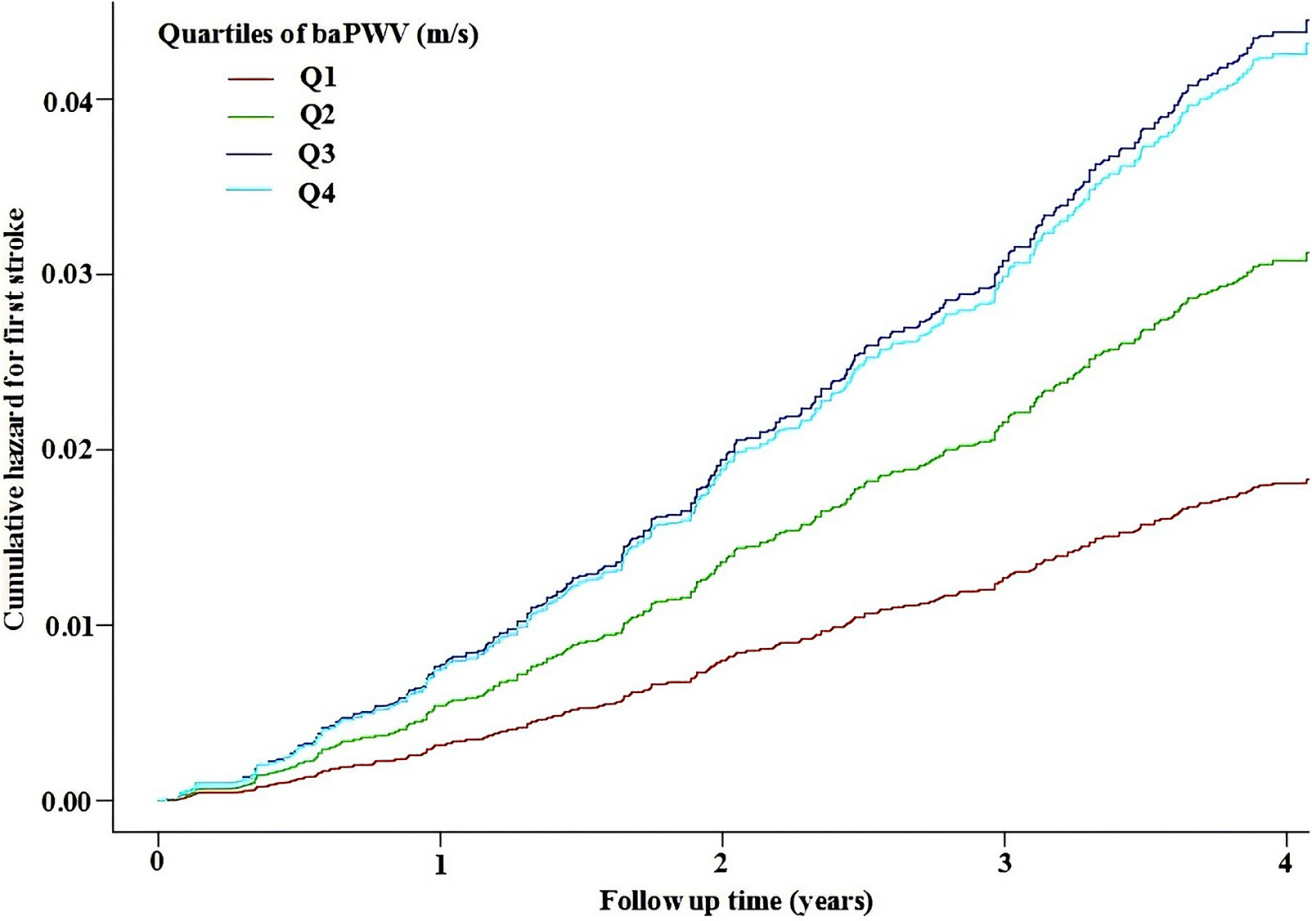
Kaplan–Meier curves for the cumulative hazard of the first stroke stratified by baPWV quartiles. Adjustment factors included gender, age, BMI, SBP, DBP, Hcy, TC, TG, HDL-C, LDL-C, current smoking, current drinking, sleep duration, antihypertensive drugs, glucose-lowering drugs, lipid-lowering drugs, antiplatelet drugs, diabetes, CHD, heart failure, and dyslipidemia. BMI, body mass index; SBP, systolic blood pressure; DBP, diastolic blood pressure; Hcy, homocysteine; TC, total cholesterol; TG, triglycerides; HDL-C, high-density lipoprotein cholesterol; LDL-C, low-density lipoprotein cholesterol; CHD, coronary heart disease.

The smoothing curve fitting revealed a saturation effect of baPWV on the first stroke (Figure 2). The saturation effect analysis revealed that the inflection point of baPWV was 17.5 m/s. For baPWV < 17.5 m/s, the adjusted HR (95% CI) was 1.31 (1.17, 1.47), and the adjusted HR (95% CI) was 0.99 (0.96, 1.02) for baPWV ≥ 17.5 m/s (*P* for log-likelihood ratio test < 0.001) (Table 3). In addition, the Kaplan–Meier curves revealed a consistent increase in cumulative hazard for the first stroke from quartile 1 to quartile 3 of baPWV (log-rank *P* < 0.001), whereas there was a non-significant difference in cumulative hazard between quartiles 3 and 4 (log-rank *P* = 0.873) (Figure 3). To verify the robustness of our findings, we also conducted a sensitivity analysis using the average of both sides, and the conclusions remained consistent with the main analysis (Supplementary Table S1 and Table S2).

**Table 3:** Saturation effect analysis of baPWV on stroke.

baPWV (m/s)	N	Events, *n* (%)	Model 1		Model 2		Model 3	
			HR (95% CI)	*P*	HR (95% CI)	*P*	HR (95% CI)	*P*
Per 1 m/s increase	7,198	281 (3.9)	1.02 (1.01, 1.04)	<0.001	1.02 (1.01, 1.04)	0.002	1.02 (1.01, 1.04)	0.025
Inflection point								
<17.5 m/s	3,824	104 (2.7)	1.37 (1.24, 1.52)	<0.001	1.36 (1.22, 1.51)	<0.001	1.31 (1.17, 1.47)	<0.001
≥17.5 m/s	3,374	177 (5.2)	0.99 (0.97, 1.02)	0.687	0.99 (0.96, 1.02)	0.627	0.99 (0.96, 1.02)	0.431
*P* for LLR test			<0.001		<0.001		<0.001	

Model 1 was adjusted for none.

Model 2 was adjusted for gender and age.

Model 3 was adjusted for gender, age, BMI, SBP, DBP, Hcy, TC, TG, HDL-C, LDL-C, current smoking, current drinking, sleep duration, antihypertensive drugs, glucose-lowering drugs, lipid-lowering drugs, antiplatelet drugs, diabetes, CHD, heart failure, and dyslipidemia.

HR, hazard ratio; CI, confidence interval; LLR, log-likelihood ratio; BMI, body mass index; SBP, systolic blood pressure; DBP, diastolic blood pressure; Hcy, homocysteine; TC, total cholesterol; TG, triglycerides; HDL-C, high-density lipoprotein cholesterol; LDL-C, low-density lipoprotein cholesterol; CHD, coronary heart disease.

### 3.4 Subgroup analyses

**Figure 4:**
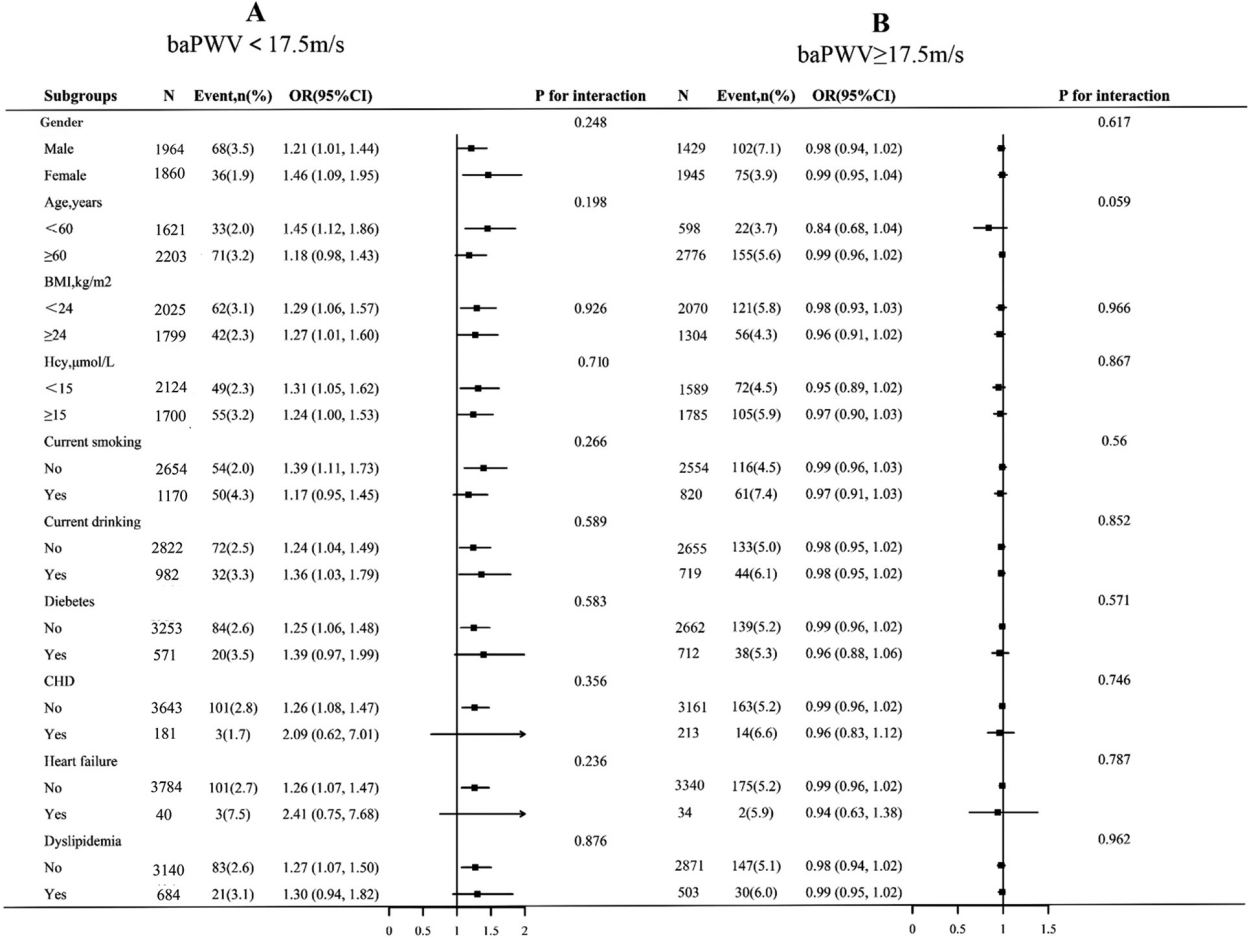
Subgroup analyses of the effect of baPWV on first stroke, grouped by baPWV < 17.5 m/s **(A)** and baPWV ≥ 17.5 m/s **(B)**. Each subgroup analysis was adjusted, if not stratified, for gender, age, BMI, SBP, DBP, Hcy, TC, TG, HDL-C, LDL-C, current smoking, current drinking, sleep duration, antihypertensive drugs, glucose-lowering drugs, lipid-lowering drugs, antiplatelet drugs, diabetes, CHD, heart failure, and dyslipidemia. BMI, body mass index; SBP, systolic blood pressure; DBP, diastolic blood pressure; Hcy, homocysteine; TC, total cholesterol; TG, triglycerides; HDL-C, high-density lipoprotein cholesterol; LDL-C, low-density lipoprotein cholesterol; CHD, coronary heart disease.

To determine the effect of covariates on the association between baPWV (per 1 m/s increment) and first stroke, stratified analyses were performed in two groups of participants separated by the inflection point of baPWV (17.5 m/s). The stratified analysis results revealed that the association of baPWV with the first stroke in various subgroups was consistent with the findings of the saturation effect analysis (Figure 4). In both the baPWV < 17.5 m/s and the baPWV ≥ 17.5 m/s groups, there were no significant interactions in the following subgroups: gender (male vs. Female), age (60 vs. ≥ 60 years), BMI (<24 vs. ≥ 24 kg/m^2^), current smokers (no vs. yes), hcy (15 vs. ≥ 15 mmol/L), current smoking (no vs. yes), current drinking (no vs. yes), diabetes (no vs. yes), CHD (no vs. yes), heart failure (no vs. yes), and dyslipidemia (no vs. yes) (*P* > 0.05 for interaction).

## 4 Discussion

This cohort study demonstrated a saturation effect with the inflection point of 17.5 m/s of baPWV on the first stroke. Higher baPWV was positively associated with a higher risk of first stroke among hypertensive adults with baPWV < 17.5 m/s.

Several studies have reported associations between baPWV and cardiovascular and cerebrovascular diseases. A Meta-analysis of 12 cohort studies revealed that baPWV is an independent predictor of cardiovascular disease, and an increase of 1 m/s was associated with a 12% increase in the risk of cardiovascular events (20). Li C et al. followed up 19,217 Chinese patients with hypertension for three years and discovered that increased baPWV was significantly associated with new-onset stroke among patients with hypertension aged < 65 years (21). Hu L et al. followed up 9,787 patients with hypertension for 20.8 months in China and demonstrated that higher baPWV levels were associated with an increased risk of first stroke (22). Kim HL et al. investigated 2,561 Korean patients with hypertension with a follow-up period of 4.14 years and discovered that increased baPWV was associated with a higher risk of future cardiovascular events (5). Kawai et al. investigated 338 Japanese patients with essential hypertension with a mean follow-up of 6.3 years and discovered that patients with higher baPWV exhibited more cardiovascular events and stroke (7). Consistent with these findings, our results revealed that an increased baPWV (<17.5 m/s) was positively associated with a higher risk of first stroke among hypertensive adults. Compared to other studies, we discovered a threshold saturation effect between baPWV and stroke rather than a linear association. Consequently, we propose that baPWV within a certain range could be an effective index for predicting future cerebrovascular events in patients with hypertension.

Several mechanisms could explain the association between baPWV and the first stroke. First, increased arterial stiffness is related to decreased local cerebral blood flow and higher cerebrovascular reactivity (23). Changes in cerebrovascular hemodynamics and arteriolar damage can cause central nervous system damage, including stroke. As baPWV levels increase, endothelial function is impaired in patients with acute stroke (24). Second, increased arterial stiffness indicates a narrowing of the peripheral arteries, which is conducive to cardiovascular disease (25).

Identifying an effective threshold value for baPWV will aid in guiding treatment strategies for cardiovascular and cerebrovascular diseases. A Japanese study proposed a cutoff value of 18.3 m/s for baPWV to predict future cardiovascular events in hypertensive populations (26). Similarly, the Japanese Circulation Society proposed a baPWV of 18 m/s as the threshold for high-risk (27). A South Korean study supported a baPWV cutoff point of 16.3 m/s for predicting cardiovascular diseases in patients with hypertension (5). In Chinese studies, baPWV cutoff values of 21.43 (22) and 20 m/s (13) were proposed to predict future cerebrovascular events. We identified a threshold of 17.5 m/s for stroke risk prediction, consistent with the findings of a Japanese cohort study (7). Variations across studies may be attributed to differences in ethnicity, study endpoints, or follow-up durations. The observed threshold effect (17.5 m/s) can be mechanistically explained as follows: First, the pathophysiological threshold of arterial remodeling; when arterial stiffness reaches a critical level, the elastic reserve of the vascular wall may be completely depleted, stabilizing hemodynamic impacts of further stiffness progression, while other risk factors (for instance, blood pressure variability, and plaque vulnerability) may become more direct triggers of stroke (28, 29). Second, cerebrovascular compensatory mechanisms (30); under severe arterial stiffness, cerebral vessels may partially compensate for hypoperfusion through autoregulation (for instance, collateral circulation recruitment), leading to slower acceleration of stroke risk (30, 31). Additionally, the inclusion of patients with hypertension with baseline vascular damage exceeding that of the general population might obscure the additional risks associated with higher baPWV (32).

Our study has several advantages, including the large sample size, the study design, and advanced statistical techniques. Moreover, to our knowledge, this is the first report of the saturation effect of baPWV on the first stroke in adults with hypertension. However, this study has several limitations. First, although a few confounding covariates were adjusted, other potential confounding effects could not be completely excluded. Second, all enrolled patients were from China, limiting the generalizability of the findings to other populations. Third, the large number of random baPWV deletions resulted in a relatively small sample size. Fourth, accurate stroke subtyping (ischemic stroke and intracerebral hemorrhage) could not be performed in some cases due to incomplete neuroimaging data (for instance, missing or low-quality CT/MRI scans), which may limit subtype-specific interpretations.

## 5 Conclusions

BaPWV exhibited a saturation effect on the first stroke in hypertensive adults in China. Increased baPWV (<17.5 m/s) was positively associated with a higher risk of first stroke among hypertensive adults. Our results demonstrate that baPWV could be an effective and simple tool for assessing first stroke in China. Intervention strategies, including intensified blood pressure control, comprehensive vascular risk management, and dynamic monitoring of baPWV, should be prioritized for patients with hypertension with elevated baPWV. If risk levels persist or escalate, further evaluation of the target organ damage is warranted.

## Data Availability

The original contributions presented in the study are included in the article/Supplementary Material, further inquiries can be directed to the corresponding author.
