# Supplementary material for: Saturation effect of brachial-ankle pulse wave velocity on first stroke in adults with hypertension: a prospective cohort study
Source: Front Cardiovasc Med. 2025 Sep 3;12:1535366. doi: 10.3389/fcvm.2025.1535366 (PMC12441068; doi:10.3389/fcvm.2025.1535366)
Supplement: Supplementary file 1 [file Datasheet1.pdf]

Table S1. Association between average baPWV and first stroke

baPWV, m/s	Model1		Model2		Model3	
	HR(95%CI)	P	HR(95%CI)	P	HR(95%CI)	P
Per 1 m/s increase	1.02 (1.01, 1.03)	<0.001	1.02 (1.01, 1.03)	0.013	1.02 (1.01, 1.03)	0.021
Quartiles						
Q1 (<15.0)	0.34 (0.23, 0.50)	<0.001	0.35 (0.23, 0.53)	0.003	0.37(0.24, 0.56)	<0.001
Q2 (15.1~17.0)	0.64 (0.46, 0.88)	0.007	0.64 (0.47, 0.89)	0.008	0.67 (0.49, 0.94)	0.002
Q3 (17.1~19.9)	1.00		1.00		1.00	
Q4 (≥20.0)	1.05 (0.79, 1.39)	0.748	1.04 (0.78, 1.39)	0.771	1.01 (0.75, 1.34)	0.987
P for trend	0.273		0.437		0.784	

Model 1 was adjusted for none.

Model 2 was adjusted for gender and age.

Model 3 was adjusted for gender, age, BMI, SBP, DBP, Hcy, TC, TG, HDL-C, LDL-C, current smoking, current drinking, sleep duration, antihypertensive drugs, glucose-lowering drugs, lipid-lowering drugs, antiplatelet drugs, diabetes, CHD, heart failure, and dyslipidemia.

Abbreviations: HR, hazard ratio; CI, confidence interval; LLR, log-likelihood ratio; BMI, body mass index; SBP, systolic blood pressure; DBP, diastolic blood pressure; Hcy, homocysteine; TC, total cholesterol; TG, triglycerides; HDL-C, high-density lipoprotein cholesterol; LDL-C, low-density lipoprotein cholesterol; CHD, coronary heart disease.

Table S2. Saturation effect analysis of average baPWV on stroke

baPWV, m/s	Model1		Model2		Model3	
	HR(95%CI)	P	HR(95%CI)	P	HR(95%CI)	P
Per 1 m/s increase	1.02 (1.01, 1.03)	<0.001	1.02 (1.01, 1.03)	0.013	1.02 (1.01, 1.03)	0.021
Inflection point						
<17.5 m/s	1.35 (1.22, 1.48)	<0.001	1.333(1.21, 1.48)	<0.001	1.29 (1.16, 1.43)	<0.001
≥17.5 m/s	0.99 (0.96, 1.02)	0.592	0.99(0.96, 1.02)	0.548	0.99 (0.96, 1.02)	0.402
P for LLR test	<0.001		<0.001		<0.001	

Model 1 was adjusted for none.

Model 2 was adjusted for gender and age.

Model 3 was adjusted for gender, age, BMI, SBP, DBP, Hcy, TC, TG, HDL-C, LDL-C, current smoking, current drinking, sleep duration, antihypertensive drugs, glucose-lowering drugs, lipid-lowering drugs, antiplatelet drugs, diabetes, CHD, heart failure, and dyslipidemia.

Abbreviations: HR, hazard ratio; CI, confidence interval; LLR, log-likelihood ratio; BMI, body mass index; SBP, systolic blood pressure; DBP, diastolic blood pressure; Hcy, homocysteine; TC, total cholesterol; TG, triglycerides; HDL-C, high-density lipoprotein cholesterol; LDL-C, low-density lipoprotein cholesterol; CHD, coronary heart disease.
