# Supplementary material for: Saturation effect of brachial-ankle pulse wave velocity on first stroke in adults with hypertension: a prospective cohort study
Source: Front Cardiovasc Med. 2025 Sep 3;12:1535366. doi: 10.3389/fcvm.2025.1535366 (PMC12441068; doi:10.3389/fcvm.2025.1535366)
Supplement: Supplementary file 2 [file Datasheet2.pdf]

Interviews are based on voluntary participation

Questionnaire for Interviews to the Surviving Participants

Guarantee for interviewee

All individual information collected in this survey will be treated as strictly confidential. The record of your name and address will be used only in future follow-up surveys to enable us to contact with you. The computerized data resulting from this survey will not include your name and address. So, nobody will be able to identify any interviewee from the computerized data files. All of the questionnaires will be stored in the locked files containers.

Questionnaire No. ____________

1. Interviewee's name:_________2. Sex: □ male □ female

3. Nationality: ____

4. Residents' health Record No.:

|__|__|__|__|__|__|__|__|__|__|__|__|__|__|__|__|__|

5. ID Card Number:

|__|__|__|__|__|__|__|__|__|__|__|__|__|__|__|__|__|__|

5.1 If your ID card indicates no birth date, please fill in your true date of birth:__________________

6. Current Address: ____

detailed village or street address (including street, apartment #, etc.)

7. Tel No: ________________ Contact person: ________________________

Tel. No. of Community Office ______ __________Person to contact at Community Office__________

8. Past disease history

(Which is the diagnostic hospital? 1= Central, municipalities and ministries affiliated hospitals 2= regional and provincial city hospitals 3= district and county hospitals 4= street, township level primary hospitals 5= health posts or private clinics)

Disease name	Yes or no<br>1 no<br>2 yes<br>9 don't know	Which is the diagnostic hospital?	Diagnosed and treatment by hospital?<br>1= no,2=yes	How long is the cumulative time?
1 Diabetes	|__|	|__|	|__|	|__||__|Year |__||__|Month
2 Stroke	|__|	|__|	|__|	|__||__|Year |__||__|Month
3 Hypertension	|__|	|__|	|__|	|__||__|Year |__||__|Month

4 Coronary heart disease	|__ |	|__ |	|__ |	|__ | |__ | Year |__| |__ | Month
4a Angina pectoris	|__ |	|__ |	|__ |	|__ | |__ | Year |__| |__ | Month
4b Myocardial infarction (MI)	|__ |	|__ |	|__ |	|__ | |__ | Year |__| |__ | Month
5 Heart failure	|__ |	|__ |	|__ |	|__ | |__ | Year |__| |__ | Month
6 Atrial fibrillation	|__ |	|__ |	|__ |	|__ | |__ | Year |__| |__ | Month
7 Dyslipidemia	|__ |	|__ |	|__ |	|__ | |__ | Year |__| |__ | Month
8 Chronic kidney disease	|__ |	|__ |	|__ |	|__ | |__ | Year |__| |__ | Month
9 Malignant tumor	|__ |	|__ |	|__ |	|__ | |__ | Year |__| |__ | Month
10 Others, please specify: __________	|__ |	|__ |	|__ |	|__ | |__ | Year |__| |__ | Month

9. Family history

Have any of your biological parents, siblings (same parents) and children been found to have the following diseases? ((1= no,2= yes 9= unknown)									
Hypertension	__	Coronary heart disease	__	Diabetes	__	Stroke	__	Malignant tumor	__(If yes, the tumor name: ________________)

10. Fracture history

1. Have you ever had a fracture?<br>1= no 2= at least 1 non-accident fracture 9= all accident fracture	|__ |
If yes, 1.1 first fracture site: 1= lumbar vertebra 2= femur 3= other parts: ______	|__ |
1.2 Age of the first occurrence (years old)	|__ | |__|

11. Medication use

Have you used antihypertensive drugs and other drugs in the past three months (a total of 7 days, excluding colds, acute diarrhea, etc.)? (Inform the patients to bring the medicine box packaging for baseline examination, each follow-up visit and physical examination.)

Type of medication	Common name (tick"√" in the ☐)	Product name or manufacturer	Dosage	Usage (times/day)	Cumulative usage time (only fill in days if it is less than one month)	Whether to bring the pill boxes 1=no 2=yes

antihypertensive drugs	Enalapril folic acid ☐			___ times daily	___ ___ ___ Month ___ ___ days	
	enalapril □			___ times daily	___ ___ ___ Month ___ ___ days	
	benazepril □			___ times daily	___ ___ ___ Month ___ ___ days	
	captopril □			___ times daily	___ ___ ___ Month ___ ___ days	
	Valsartan □			___ times daily	___ ___ ___ Month ___ ___ days	
	Losartan □			___ times daily	___ ___ ___ Month ___ ___ days	
	irbesartan □			___ times daily	___ ___ ___ Month ___ ___ days	
	Micardis □			___ times daily	___ ___ ___ Month ___ ___ days	
	amlodipine □			___ times daily	___ ___ ___ Month ___ ___ days	
	nifedipine □			___ times daily	___ ___ ___ Month ___ ___ days	
	nitrendipine □			___ times daily	___ ___ ___ Month ___ ___ days	
	Nimodipine □			___ times daily	___ ___ ___ Month ___	
	bisoprolol □			___ times daily	___ ___ ___ Month ___ ___ days	
	metoprolol □			___ times daily	___ ___ ___ Month ___ ___ days	
	indapamide □			___ times daily	___ ___ ___ Month ___ ___ days	
	DCT □			___ times daily	___ ___ ___ Month ___ ___ days	
	triamterene □			___ times daily	___ ___ ___ Month ___ ___ days	
	spirolactone □			___ Times	___ ___ ___ Month ___ ___ days	
	reserpine □			___ times daily	___ ___ ___ Month ___ ___ days	
	Jane chrysanthemum piece □			___ Times	___ ___ ___ Month ___ ___ days	
	Robuum antihypertensive tablet □			___ times daily	___ ___ ___ Month ___ ___ days	
	Beijing antihypertensive spirit □			___ times daily	___ ___ ___ Month ___ ___ days	
	other_____□			___ times daily	___ ___ ___ Month ___ ___ days	
	other_____□			___ times daily	___ ___ ___ Month ___ ___ days	
	other_____□			___ times daily	___ ___ ___ Month ___ ___ days	

Hypoglycemic drugs	melbine □			___ times daily	___ ___ ___ Month ___ days	
	acarbose □			___ times daily	___ ___ ___ Month ___ days	
	gliclazide □			___ times daily	___ ___ ___ Month ___ days	
	glimepiride □			___ times daily	___ ___ ___ Month ___ days	
	glipizide □			___ times daily	___ ___ ___ Month ___ days	
	glibenclamide □			___ times daily	___ ___ ___ Month ___ days	
	rosiglitazone □			___ times daily	___ ___ ___ Month ___ days	
	insulin □			___ times daily	___ ___ ___ Month ___ days	
	other □			___ times daily	___ ___ ___ Month ___ days	
	other □			___ times daily	___ ___ ___ Month ___ days	
	other □			___ times daily	___ ___ ___ Month ___ days	
Lipid lowering drugs	Simvastatin □			___ times daily	___ ___ ___ Month ___ days	
	atorvastatin □			___ times daily	___ ___ ___ Month ___ days	
	Rosuvastatin □			___ times daily	___ ___ ___ Month ___ days	
	fenofibrate □			___ times daily	___ ___ ___ Month ___ days	
	bezafibrate □			___ times daily	___ ___ ___ Month ___ days	
	Blood fat kang □			___ times daily	___ ___ ___ Month ___ days	
	2-dimerthylpentanoic acid □			___ times daily	___ ___ ___ Month ___ days	
	other □			___ times daily	___ ___ ___ Month ___ days	
	other □			___ times daily	___ ___ ___ Month ___ days	
	other □			___ times daily	___ ___ ___ Month ___ days	
Antiplatelet/ anticoagulant drugs	aspirin □			___ times daily	___ ___ ___ Month ___ days	
	clopidogrel □			___ times daily	___ ___ ___ Month ___ days	
	warfarin □			___ times daily	___ ___ ___ Month ___ days	
	other_____ □			___ times daily	___ ___ ___ Month ___ days	
	other □			___ times daily	___ ___ ___ Month ___ days	
	other_____ □			___ times daily	___ ___ ___ Month ___ days	
vitamins	B vitamins □			___ times daily	___ ___ ___ Month ___ days	
	D group of vitamins □			___ times daily	___ ___ ___ Month ___ days	
	vitamin complex □			___ times daily	___ ___ ___ Month ___ days	

	Others, please specify: ________			|__ |times daily	|___| |___	|___	Month |___			days	
Chinese traditional medicine	Danshen drop pill □			|__ |times daily	|___| |___	|___	Month |___			days	
	Tongxinluo capsule, □			|__ |times daily	|___| |___	|___	Month |___			days	
	Stable heart particles □			|__ |times daily	|___| |___	|___	Month |___			days	
	suxiao jiuxin pills □			|__ |times daily	|___| |___	|___	Month |___			days	
	Liuwei Dihuang Wan □			|__ |times daily	|___| |___	|___	Month |___			days	
	Thromboone capsule □			|__ |times daily	|___| |___	|___	Month |___			days	
	Others, please specify: ________			|__ |times daily	|___| |___	|___	Month |___			days	
	Shenmai Injection □				__	times daily		_	Times / year	
	Hematatone_______□ injection			|__ |times daily	Accumulated	__		__	times	
	Other injection______□				__	times daily		_	Times / year	
	solution, __					
	Others, please specify: ________			|__ |times daily	Accumulated	__		__	times	

Adverse events that occurred after taking statins 1=no 2=yes, if no, skip the following options

1. Gastrointestinal symptoms: □ Fatigue □ Loss of appetite □ Hatred of oil □ Distension and pain in liver area □ Upperabdominal discomfort, etc

2. Manifestations of portal hypertension : □ Ascites □ Jaundice □ Hepatomegaly □ Varicose veins

3. muscle injuries: □ Myalgia □ Muscle weakness □ Muscle spasm □ Weakness □ Fatigue □ Fever □ osteoarthralgia

4. biochemical examination abnormalities:

□ ALT or TBil single item>2 times ULN

□ ALT, ALP and TBil simultaneously>ULN and at least of them>2 times ULN

□ Ck>5 times ULN

□ Myoglobin >ULN

If there is any abnormality, collect the test data results:.

ULN:______; ALT:______; TBIL:______; ALP:______;CX:______; myoglobin:______

The above adverse events, if any,

1,Is there any other reasonable explanation: 1=no,2=yes

2. Will it lead to drug-discontinuation or dose reduction?1=no,2=yes

12. career and living conditions

1. The nature of your work unit (fill in the work unit where you worked before retirement if you are retired):<br>1= administrative institution 2= large enterprises or companies (more than 300 people) 3= small business or self-employed people 4= farmers 5= other ( )	|__|
2, the current specific occupation (if currently engaged for less than 1 year, ask about the previous occupation for more than 1 year):<br>1= agriculture, forestry, animal husbandry,fishery, water conservancy production personnel<br>2= production, transportation equipment operatorsand related personnel<br>3= business, service personnel<br>4= head of state agencies, party and mass organizations,enterprises, public institutions<br>5= service staff and related personnel 6= professional and technical personnel<br>7= military 8= other workers 9= school students 10= unemployed 11= housework<br>12= retired and no longer working	|__||__|
3. What is your education level (note: the diploma recognized by the state shall prevail)<br>1= illiterate 2= self-study or private school<br>3= not graduated from primary school 4= graduated form primary school 5= graduated from junior high school<br>6= graduated from high school / technical secondary school / technical school<br>7= graduated form college 8=bachelor's degree 9= graduate student or above	|__|
3, your local living standard is in: 1= better 2= average 3= deviation	|__|
4. How intense is the physical labor in your daily work?1= light, 2= medium, 3= heavy	|__|
5. Does your main occupation and your daily life cause you psychological stress?<br>1= relaxed and not nervous 2= nervous 3= very nervous and stressed	|__|

6. How is your usual sleep quality at night?1= good, 2= average 3= poor	|__|
6.1 Do you have the nap habit?1= no 2= Yes	|__|
7. How long do you sleep for a day on average?1=5 hours 2 = 5 to 8 hours 3= 8 hours	|__|
7.1 Do you snore in your sleep?1= rarely or never 2= frequent snoring, but not heavy 3= loud snoring, sometimes makes you awake	|__|
8, are you left-handed or right-handed 1= left 2= right 3= left, but not completely	|__|
9, your current marital status is: 1= single 2= in marriage 3= divorced 4= widowed 5=others	|__|

13. Smoking status (smoking refers to at least one cigarette per day for more than one year, or more than 18 packs per year)

1. Do you currently smoke?**1= No (jump question 2) 2= Yes;**	|__|
. 1If so, what age did you start smoking?(years old)	|__||__|
1.2 How many cigarettes do you smoke on an average day?(conversions will be made for incomplete cigarettes smoking)	|__||__|
1.2.1 How many years have you been smoking like this?	|__||__|
1.2.2 Main types of cigarettes 1= cigarette 2= roll-your-own cigarettes 3= tobacco bag (hookah) 4= Other: ___<br>After answering, skip to drinking	|__|
2. Have you ever smoked cigarettes?'1=no (ask about drinking) 2=yes	|__|
2.1 If yes, at what age did you start smoking ?(years old)	|__||__|
2.2 How many cigarettes did you smoke per day on average at that time ?(conversions will be made for incomplete cigarettes smoking)	|__||__|
2.2.1 How many years had you been smoking like that?	|__||__|
2.2.2 Main types of cigarettes 1= cigarette 2= roll-your-own cigarettes 3= tobacco bag (hookah) 4= Other: ___	|__|
2.3 At what age did you stop smoking?(years old)	|__||__|
2.3. 1 Your reasons for quitting smoking 1= health reasons 2= economic reasons<br>3= recognize that smoking is harmful 4= family objections 5= Others(<br>____)	|__|

14. Drinking (drinking refers to drinking twice or more times per week for more than 1 year)

1. Do you currently drink alcohol?**1= No (jump question 2) 2= Yes**	|__|
1.1 At what age did you start drinking? (years old)	|__||__|
1.2 What kind of wine do you often drink?1= No;2= Yes	
1.2.1 | __ | white wine 1.2.2 | __ | wine (red wine) 1.2.3 | __ | __ | beer 1.2.4 | __ | rice wine or yellow rice wine 1.2.5 | __ | __ | other: _______	
1.3 How much wine do you drink every week?	
1.3. 1 liquor | __ | | __ | ounce 1.3.2 wine | __ | | __ |ounce 1.3.3 beer | __ | | | __<br>bottles .3.41 rice wine or rice wine | | | | __ | ounce 1.3.5 other: _____<br>After answering, skip to diet situation	
2. Have you ever drank alcohol?1=NO(skip the question) 2=yes	
2.1 At what age did you start drinking alcohol (years old)	
2.2 What wine you used to drink?1= No;2= Yes	
2.2.1 | __ | liquor 2.2.2 | __ | wine (red wine) 1.2.3 | __ | beer 1.2.4 | __ | rice wine or yellow rice wine<br>1.2.5 | __ | Other: _______	
1.3 How much alcohol did you drink every week?	

1.3.1 liquor	__		__	ounce 1.3.2 wine	__		__	ounce 1.3.3 beer	__			__ bottles .3.41 rice wine or rice wine				__ |ounce 1.3.5 other: ______	
If not, 1.4.2 At what age did you stop drinking?Age ______________ years (years old)	
1.4.2.1 The reasons for quit drinking are 1= health reasons 2= economic reasons 3= recognize that drinking is harmful 4= family objections 5= other (________________________________)	

15. Diet conditions

<table>
<tr><td>1. What is your main staple food?1= rice, 2= pasta 3= other (______________)</td><td>|__|</td></tr>
<tr><td>2. Your average daily amount of staple food (gram)</td><td>|__||__|</td></tr>
<tr><td>3. Cooking oil
1= complete vegetable oil 2=main vegetable oil 3= basically half and half
4= main animal oil</td><td>|__|</td></tr>
<tr><td>4. How many times do you eat tofu per week on average?
1= less than once or never 2=1-2times 3=3-5times 4= almost every day</td><td>|__|</td></tr>
<tr><td>5. How many times a week do you eat meat (chicken, duck, fish, pigs, cattle, sheep, etc.)
1= less than once or never 2=1-2times 3=3-5times 4= almost every day</td><td>|__|</td></tr>
<tr><td>6. What kind of meat do you often eat?
1=no meat 2= lean meat mainly 3=fat and lean 4=fat mainly</td><td>|__|</td></tr>
<tr><td>7, how much fruit and vegetable do you eat per week on average?
1= less than 1 catties 2=1kg-3kg 3=more than 3 pounds</td><td>|__|</td></tr>
<tr><td>8, Do your meals taste light(bland)or heavy(salty)?
1= light 2= average 3= heavy</td><td>|__|</td></tr>
<tr><td>9. Do you often take vitamin supplements?
1= no supplement 2= 1-2 times / week 3=3-5 times / week 4= almost every day</td><td>|__|</td></tr>
<tr><td>9. 1 If you supplement the vitamin, the name of the vitamin is________</td><td></td></tr>
</table>

16. Female menstruation and reproductive history

<table>
<tr><td>1. At what age did you have your first menstruation?</td><td>|__||__|</td></tr>
<tr><td>2. How long does your period usually last on average?(days)</td><td>|__||__||__|</td></tr>
<tr><td>3. Is your menstruation regular?1= No,2= Yes</td><td>|__|</td></tr>
<tr><td>4. Do you have dysmenorrhea during menstruation?1= No,2= Yes</td><td>|__|</td></tr>
<tr><td>4.1 If you have dysmenorrhea, is it serious?1= light, 2= medium, 3= heavy</td><td>|__|</td></tr>
<tr><td>Are you menopausal? 1= No(skip to question 6) ,2= Yes</td><td>|__|</td></tr>
<tr><td>5.1 At what time did you go through menopause?</td><td>|__||__|</td></tr>
<tr><td>5.2 Do you use oestrogen replacement therapy (> half a year)?1= No,2= Yes</td><td>|__|</td></tr>
<tr><td>6. Are you in your period?1= No,2= Yes</td><td>|__|</td></tr>
<tr><td>7. Do you take birth control pills (> half a year)?1= No,2= Yes</td><td>|__|</td></tr>
<tr><td>8. How many times have you ever been pregnant?(Including all miscarriages, induced labour, live births)</td><td>|__||__|</td></tr>
<tr><td>9. Have you ever had an induced abortion?(Write the number of times, never= 0)</td><td>|__|</td></tr>
<tr><td>10. Have you ever had a spontaneous abortion?(Write the number of times, never=0)</td><td>|__|</td></tr>
</table>

17. Consultation room blood pressure (subjects should sit quietly and rest for at least 15 minutes)

17.1 Time since the last antihypertensive drug was taken (999= more than 7 days): |__| |__| |__| hours

17.2 If it has been more than 7 days since the last dose, explain the reason: ____ .

17.3 Sitting blood pressure on the day, measurement time:_______________

Right arm	
Blood pressure value (sitting position) (Systolic /Diastolic mmHg)	Pulse (beats /min)
First measurement ______/_______	______
Second measurement______/_______	______
Third measurement____/_______	______
Fourth measurement______/_______	______

18, You have had a history of hypertension for ____ years, the highest blood pressure has reached: _______ / ______mmHg.

19. Height: _____.___ cm; Weight: ______.___ kg;

Waist Circumference ___.__ cm; Hip circumference, _____.__ cm
